# Chronic Spontaneous Urticaria After COVID-19 Vaccine

**DOI:** 10.7759/cureus.18102

**Published:** 2021-09-19

**Authors:** John Thomas, George Thomas, Ajay Chatim, Param Shukla, Matthew Mardiney

**Affiliations:** 1 N/A, West Virginia School of Osteopathic Medicine, Lewisburg, USA; 2 N/A, George Washington University School of Medicine and Health Sciences, Washington, USA; 3 N/A, New York Institute of Technology College of Osteopathic Medicine, Old Westbury, USA; 4 N/A, Rutgers New Jersey Medical School, Newark, USA; 5 Allergy and Immunology, University of Maryland St. Joseph Medical Center, Towson, USA

**Keywords:** chronic, spontaneous urticaria, covid-19 vaccine complication, pfizer-biontech vaccine, erythematous plaques, hives

## Abstract

Public health efforts over the past few months have been aimed at vaccinating young adults. Moderna and Pfizer COVID-19 vaccines are widely available options. Cutaneous reactions to these vaccines have been described as self-limiting and relatively immediate after vaccine administration. In this case report, we present a young adult who received the Pfizer-BioNTech BNT162b2 mRNA COVID-19 vaccine and developed chronic, spontaneous urticaria.

## Introduction

As many states are relaxing their COVID-19 pandemic restrictions, concerted public health efforts have focused on increasing vaccination rates in the country. Pfizer and Moderna are leading the vaccine charge with their own vaccines which were approved by the Food and Drug Administration (FDA) via emergency authorization in December 2020. Since then, millions of people across the United States have received vaccines in an effort to return to normalcy. As with any drug or vaccine, the COVID-19 vaccine has also recorded many adverse reactions. However, most of the recorded cases have been of myocarditis and pericarditis in young adults after the second dose [1]. Not many cutaneous reactions have been described; therefore, the goal of this article is to document a case of urticarial rash after the patient received the second dose of the Pfizer vaccine.

Some studies have illustrated the variety of dermatological allergic reactions from the COVID-19 vaccine. One study reported that among hypersensitive reactions, delayed reactions were less frequently observed than immediate reactions [2]. While none of the studies have mentioned the same reaction observed in this case report, a few of them have reported similar cutaneous reactions. One report observed that 414 patients had delayed, large, local cutaneous reactions from the COVID-19 vaccine [3]. Another report outlined that 16 people had similar allergic reactions after the Moderna vaccine [4]. However, all reports published had a short self-limited resolution to the cases presented, with no chronic cases observed.

While studies reporting the side effects of the various vaccines have been published, we present here the first reported case, to our knowledge, of a patient with a chronic spontaneous urticarial rash delayed one week after receiving the second dose of the Pfizer-BioNTech BNT162b2 mRNA COVID-19 vaccine timely on Day 21. Comprehensive blood tests, skin allergy testing, physical exam, and patient medical history were fully scoped. With no associated allergic reactions to vaccinations or other relevant substances, the patient continues to suffer from chronic, universal, and random body rashes daily with no improvement.

## Case presentation

A male patient in his early 20s presented to the allergy clinic eight weeks after receiving his second dose of the Pfizer-BioNTech BNT162b2 mRNA COVID-19 vaccine in March 2021 for a recurrent rash.

The patient had no chronic medical conditions and had not been diagnosed with COVID-19 in the past. The patient’s vaccination history was up to date with no prior allergic reactions. The patient has had a severe allergy to sulfa drugs since two years old with a kidney failure reaction but was not taking any prescribed or over-the-counter medications. 

**Figure 1:**
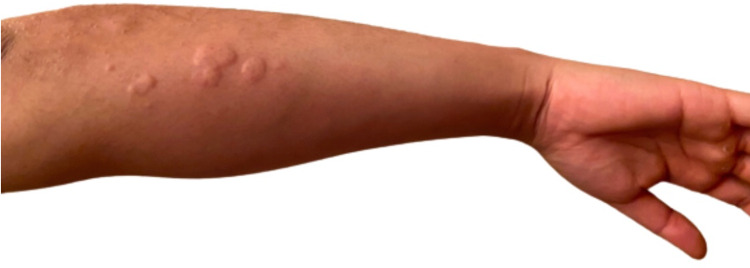
Maculopapular Urticaria on Left Forearm Chronic spontaneous maculopapular urticaria on left forearm with raised, pruritic erythematous plaques that have presented daily for 30-60 minutes for the past eight weeks in widespread global hive formation.

The patient had no immediate reactions to the second dose of the vaccine, except for arm soreness which resolved in 24 hours. One week later, the patient developed a global widespread urticarial formation of a daily rash with maculopapular wheels. The rash would disappear after 30-60 minutes and reappear one to two hours later or the next day at a different random location (Figure 1). The patient had no other associated symptoms such as angioedema or shortness of breath and denied any changes to diet or environmental stimuli, including no exposure to any pets or animals, over the past six months.

The rash would resolve within 15 minutes of oral Zyrtec administration but reappear again globally throughout the body within 24 hours after taking a Zyrtec dose. The patient has tried taking Zyrtec consistently for various prolonged periods of time, but urticaria would resume after 24 hours of ceasing daily dosage. Complete blood count and comprehensive metabolic panel were unremarkable. Thorough allergy skin patch testing showed mild reactivity to walnuts and sesame seeds, which are not included in the patient's daily diet. No reactivity was shown to shellfish, seafood, or any other possibly relevant substances. Currently, the patient still has the rash daily. Physical exam and patient history indicate rash is chronic spontaneous urticarial.

## Discussion

According to the Centers for Disease Control and Prevention (CDC), only select patients within the appropriate age range (individuals 12 and above) should not receive the COVID-19 vaccine. Patients with a known severe allergic reaction to any of the ingredients found in the vaccine, should not receive either dose. Also, patients that experienced a severe or immediate allergic reaction after getting the first dose, should not receive the second dose [1]. The patient described in the case above did not qualify for either of these exceptions.

Prior studies have documented cutaneous reactions to the COVID-19 vaccine. Stingeni et al. reported six patients that developed mucocutaneous allergic reactions after the first dose of the Pfizer-BioNTech vaccine that self-resolved [2]. Freeman et al. reported varying cutaneous reactions in 414 patients to mRNA COVID-19 vaccines, most of which were delayed large self-limited, local reactions [3]. Little et al. reported 16 patients with delayed local cutaneous reactions to the Moderna vaccine that resolved in a median time of 21 days [4].

## Conclusions

In this case report, we present a male patient in his early 20’s with chronic spontaneous urticarial rashes after receiving the second dose of the COVID-19 Vaccine (Pfizer Vaccine). These rashes appear to be raised erythematous maculopapular wheels that are pruritic that present daily for 30-60 minutes in random locations globally throughout the body. The rashes resolve within 15 minutes of oral Zyrtec administration. Our case report is the first, to our knowledge, to report a patient that developed a chronic spontaneous urticarial rash after the second dose of the vaccine that is not self-limited. CDC has been made aware of this unique long-term reaction. Further investigations are prompted to find other patients that may have similar reactions to the mRNA COVID-19 vaccine and to provide awareness for potential chronic reactions to the vaccine.
